# miR-34a as hub of T cell regulation networks

**DOI:** 10.1186/s40425-019-0670-5

**Published:** 2019-07-16

**Authors:** Martin Hart, Barbara Walch-Rückheim, Lena Krammes, Tim Kehl, Stefanie Rheinheimer, Tanja Tänzer, Birgit Glombitza, Martina Sester, Hans-Peter Lenhof, Andreas Keller, Eckart Meese

**Affiliations:** 1 Institute of Human Genetics, Saarland University, Building 60, 66421 Homburg, Germany; 2 Institute of Virology and Center of Human and Molecular Biology, Saarland University, 66421 Homburg, Germany; 3 Center for Bioinformatics, Saarland University, 66123 Saarbrücken, Germany; 4 Department of Transplant and Infection Immunology, Saarland University, 66421 Homburg, Germany; 5 Chair for Clinical Bioinformatics, Saarland University, 66123 Saarbrücken, Germany

**Keywords:** miR-34a, Immune system process, CD11A, VAMP2, CD4 + & CD8^+^ T cells

## Abstract

**Background:**

Micro(mi)RNAs are increasingly recognized as central regulators of immune cell function. While it has been predicted that miRNAs have multiple targets, the majority of these predictions still await experimental confirmation. Here, miR-34a, a well-known tumor suppressor, is analyzed for targeting genes involved in immune system processes of leucocytes.

**Methods:**

Using an *in-silico* approach, we combined miRNA target prediction with GeneTrail2, a web tool for Multi-omics enrichment analysis, to identify miR-34a target genes, which are involved in the immune system process subcategory of Gene Ontology.

**Results:**

Out of the 193 predicted target genes in this subcategory we experimentally tested 22 target genes and confirmed binding of miR-34a to 14 target genes including *VAMP2*, *IKBKE*, *MYH9*, *MARCH8*, *KLRK1*, *CD11A*, *TRAFD1*, *CCR1*, *PYDC1*, *PRF1*, *PIK3R2*, *PIK3CD*, *AP1B1*, and *ADAM10* by dual luciferase assays. By transfecting Jurkat, primary CD4^+^ and CD8^+^ T cells with miR-34a, we demonstrated that ectopic expression of miR-34a leads to reduced levels of endogenous VAMP2 and CD11A, which are central to the analyzed subcategories. Functional downstream analysis of miR-34a over-expression in activated CD8^+^ T cells exhibits a distinct decrease of PRF1 secretion.

**Conclusions:**

By simultaneous targeting of 14 mRNAs miR-34a acts as major hub of T cell regulatory networks suggesting to utilize miR-34a as target of intervention towards a modulation of the immune responsiveness of T-cells in a broad tumor context.

**Electronic supplementary material:**

The online version of this article (10.1186/s40425-019-0670-5) contains supplementary material, which is available to authorized users.

## Background

To profoundly decipher the molecular mechanisms of the immune response it is crucial to investigate the role of miRNAs in the regulation of T cells. Micro(mi)RNAs, which are small non coding RNAs of ~ 21–24 nucleotides in length, play a crucial role in regulating gene expression post-transcriptionally [1]. Specifically, miRNAs inhibit protein biosynthesis by binding to sequences in 3′ untranslated regions (3’UTR) or in fewer instances in 5′ untranslated regions or open reading frames of their target mRNA [2, 3]. While changes in disease related miRNA expression have extensively been studied in various cancer types [4], it is likewise important to explore molecular functions of deregulated miRNAs in immune cells of tumor patients. Previously, we analyzed the miRNA expression of whole blood samples from patients with various types of cancer including prostate cancer, lung cancer, pancreatic ductal adenocarcinoma, melanoma, ovarian cancer, gastric tumors, Wilms tumor and pancreatic tumors [5–9]. In contrast to miRNA profiles obtained from human serum, the analysis of miRNAs in blood cells frequently allows tracing the origin of an altered miRNA back to a specific cell type. By analyzing the miRNA expression in different blood cell subtypes of healthy controls versus lung cancer patients we found a significant overexpression of the tumor suppressor miR-34a in CD3^+^ T cells of lung cancer patients [10]. Utilizing a dual luciferase approach for target identification [11–13], we identified five protein kinase C family members including *PRKCA*, *PRKCB*, *PRKCE*, *PRKCH* and *PRKCQ* as direct target genes of miR-34a [14]. These PKC isozymes control cell signaling through the immunological synapse downstream of the T-cell receptor (TCR) and T cell migration [15–17] further indicating a relevance of miR-34a in T cell functions. Recently, we clarified the functional role of miR-34a in the modulation of intracellular calcium signaling and NF-κB signaling [18–20]. Here, we show that miR-34a simultaneously controls the translation of mRNAs that are crucial for T cell regulatory networks.

## Methods

### Cell lines, tissue culture

The human HEK 293 T and Jurkat cells were obtained from the German collection of microorganisms and cell cultures (DSMZ) and authenticated using STR DNA typing. HEK 293 T and Jurkat cells were cultured as described previously [14]. All cell lines were cultured for less than six month after receipt.

### CD4^+^ T cell isolation and flow cytometry

CD4^+^ and CD8^+^ T cells were isolated by negative selection, purity was confirmed by flow cytometry (Additional file 1: Figure S2) and cultured in RPMI 1640 medium as mentioned earlier [20].

### Transfection of Jurkat, CD4^+^ T cells and CD8^+^ T cells

2.5 × 10^5^ Jurkat cells/2 ml/6well or 1 × 10^6^ CD4^+^ T cells/ml/12well or 1 × 10^6^ CD8^+^ T cells/ml/12well were transfected with 150 ng hsa-miR-34a-5p miScript miRNA mimic (MIMAT0000255: 5’UGGCAGUGUCUUAGCUGGUUGU), or the allstars negative control (ANC) using HiPerFect transfection reagent (Qiagen, Hilden, Germany). 48 h post transfection, cells were harvested and whole cell extracts were prepared as described above and the CD4^+^ and CD8^+^ T cells were stained with anti-CD4-FITC (RPA-T4, BD), with anti-CD8-FITC (RPA-T8, BD) and anti-CD11A-APC (HI111, BD), or respective conjugated isotype control antibodies, fixed in 1% paraformaldehyde and analyzed by flow cytometry (FACS canto II, BD biosciences)

### Dual luciferase reporter assays

For the dual luciferase reporter assays 7 × 10^4^ HEK 293 T cells per well of a 24-well plate were transfected with 200 ng/well reporter vector and 800 ng/well miR-34a expression plasmid using PolyFect transfection reagent (Qiagen, Hilden, Germany) corresponding to manufacturer’s protocol. Dual Luciferase assays were performed as mentioned earlier and according the manufacturer’s protocol [14]. For analysis the luciferase activity of each wild type 3’UTR reporter construct cotransfected with miR-34a was normalized to the luciferase activity of the empty reporter vector cotransfected with miR-34a.

### Western blot

For Western Blot analysis of CD11A and VAMP2 Jurkat, CD4^+^ T cells, or CD8^+^ T cells were transfected as described above. 48 h post transfection cells were lysed with 2x lysis buffer (130 mM Tris/HCl, 6% SDS, 10% 3-Mercapto-1,2-propandiol, 10% glycerol) and 3 times treated with ultrasound for 2 s. 15 μg of the whole protein extracts were separated by SDS gel electrophoresis in a Mini-Protean® TGX Precast Gel (Bio-Rad Laboratories Inc., Hercules, California, USA) and transferred to a nitrocellulose membrane (Whatman, GE Healthcare, Freiburg, Germany). CD11A was detected by a purified mouse anti human CD11A antibody (610826,BD, Franklin Lakes, USA), VAMP2 by a monoclonal rabbit anti human VAMP2 antibody (D601A, Cell Signaling Technology, Danvers, United States). GAPDH and β-actin were used as loading controls and detected with a monoclonal antibody against human GAPDH (14C10, Cell Signaling Technology, Danvers, United States) and an anti-β-actin monoclonal mouse antibody (AC-15, Sigma Aldrich, Munich, Germany), respectively. All secondary antibodies were obtained from Sigma Aldrich (Sigma Aldrich, Munich, Germany).

### Plasmids

The pSG5-miR-34a expression vector was generated by Eurofins Genomics containing the nucleotides 9151617–9151816 of chromosome 1 (Eurofins Genomics, Ebersberg, Germany). The 3’UTRs of *VAMP2*, *IKBKE*, *MYH9*, *MARCH8*, *KLRK1*, *CD11A*, *TRAFD1*, *CCR1*, *PYDC1*, *PRF1*, *PIK3R2*, *PIK3CD*, *AP1B1*, *ADAM10*, *PVR*, *AP2S1*, *BAD*, *ICOS*, *CD247*, *ZFP36*, *STX8* and *SPN*, were cloned into the pMIR-RNL-TK vector, which was described in Beitzinger et al. using the SpeI, SacI or NaeI restriction sites [21]. All insert were PCR amplified using specific primers and all predicted hsa-miR-34a-5p target sites of selected target genes were mutated by site-directed mutagenesis with the QuickChange II Site-Directed Mutagenesis Kit (Agilent Technologies, Santa Clara, United States) using specific primers. The identifiers of all cloned 3’UTR sequences and the sequences of specific cloning primers are shown in Additional file 1: Table S1.

### RNA-isolation, quantitative real time PCR (qRT-PCR)

The RNA isolation of ANC or miR-34a transfected CD4^+^ T cells was carried out 48 h post transfection using the miRNeasy Mini Kit corresponding to the manufacturer’s protocol (Qiagen, Hilden, Germany). The expression of hsa-miR-34a-5p, was analyzed applying qRT-PCR with the StepOnePlus Real-Time PCR System (Applied Biosystems, Foster City, United States) and the miScript PCR System (Qiagen, Hilden, Germany) according to the manufacturer’s manual. In brief, 200 ng total RNA was reverse transcribed into cDNA using the miScript RT II Kit with the miScript HiFlex Buffer (Qiagen, Hilden, Germany). RNU48 served as endogenous control for miRNA expression. Over-expression of miR-34a in the transfected CD4^+^ T cells is shown in Additional file 1: Figure S1.

### Quantification of PRF1 production by ELISA

1 × 10^6^ CD8^+^ T cells/ml/12well were transfected with 150 ng hsa-miR-34a-5p miScript miRNA Mimic (MIMAT0000255: 5’UGGCAGUGUCUUAGCUGGUUGU), or the allstars negative control (ANC) using HiPerFect transfection reagent (Qiagen, Hilden, Germany). 48 h post transfection the transfected CD8^+^ T cells were activated by PMA/Ionomycin. 4 h after activation the supernatants were collected and PRF1 quantification was performed according to the manual of the human Perforin ELISA Kit (#PK-EL-68242, PromoCell GmbH, Heidelberg, Germany).

### Data analysis

Statistical analysis of the luciferase assays, the Western Blots, the FACS analysis and ELISA was performed with SigmaPlot 10 (Systat, Chicago, USA) applying Student’s t-test. Quantification of the Western blots was carried out with Image Lab Software Version 5.2.1 (Bio-Rad Laboratories Inc., Hercules, California, USA).

## Results

### Prediction of miR-34a target genes related to T cell function

**Fig. 1:**
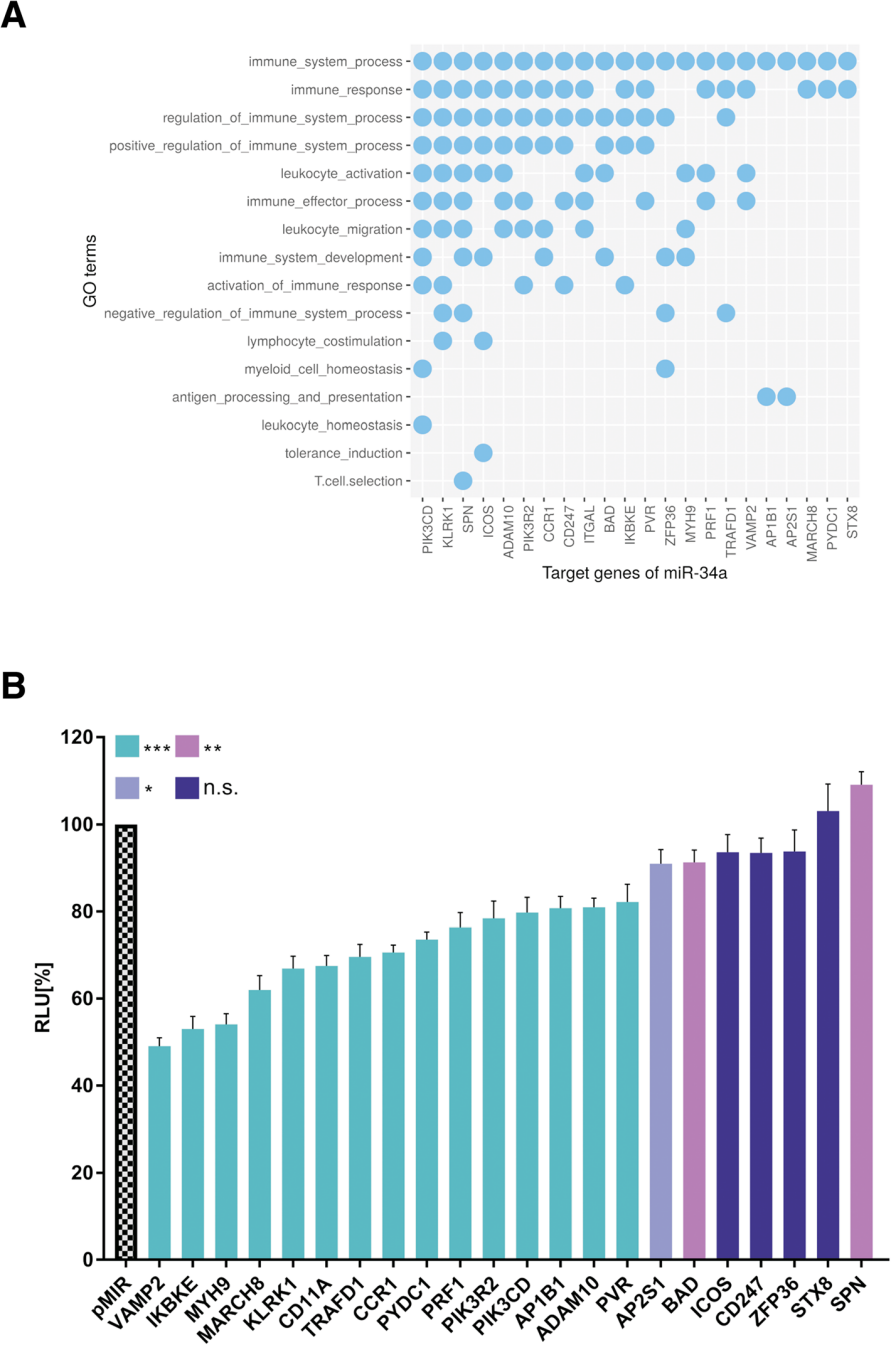
Gene Ontology subcategories of the predicted miR-34a target genes. **a** Enrichment of predicted miR-34a target genes in specific Gene Ontology subcategories. **b** Dual luciferase reporter gene assay of *VAMP2, IKBKE, MYH9, MARCH8, KLRK1, CD11A, TRAFD1, CCR1, PYDC1, PRF1, PIK3R2, PIK3CD, AP1B1, ADAM10, PVR, AP2S1, BAD, ICOS, CD247, ZFP36, STX8* and *SPN*. HEK 293 T cells were co-transfected with miR-34a and reporter plasmids containing 3’UTRs of target genes as indicated. The luciferase activities were normalized with respect to the luciferase activity measured with empty reporter construct. The results represent the mean of four independent experiments carried out in duplicates. Columns colored in turquois represent a significant reduction of the luciferase activity with a *p*-value ≤0.001 (three asterisks). Columns colored in magenta represent a significant reduction of the luciferase activity with a *p*-value ≤0.01and ≥ 0.001 (two asterisks). Columns colored in violet represent a significant reduction of the luciferase activity with a *p*-value ≤0.05 and ≥ 0.01 (one asterisk). Columns colored in dark blue represent a non-significant reduction of the luciferase activity with a *p*-value ≥0.05. Data are represented as mean ± SEM

Previously, we identified miR-34a as modulator of intracellular calcium signaling and NF-κB signaling in CD4^+^/CD8^+^ T cells [19, 20]. To investigate the overall importance of miR-34a in T cell regulation we performed an in silico prediction of target genes of miR-34a using miRWalk 2.0 [22] and identified 18828 potential target genes of miR-34a. miRWalk 2.0 combined 10 algorithms including DIANAmT, miRanda, miRDB, miRWalk, RNAhybrid, PICTAR4, PICTAR5, PITA, RNA22 and Targetscan. By including only genes that were predicted by at least 4 different target prediction algorithms, we reduced the number of target genes to 3179. To arrange the predicted target genes in pathways we used GeneTrail2 (https://genetrail2.bioinf.uni-sb.de/), a web service allowing the integrated analysis of transcriptomic, miRNomic, genomic and proteomic datasets [23]. We identified 1227 significant subcategories (*p* value ≤0.05) in Gene Ontology. We analyzed all subcategories for immune system related pathways and found the highest number of predicted miR-34a target genes in the subcategory immune system process with 193 predicted miR-34a target genes that were significantly enriched in this pathway (p value ≤0.05) (Additional file 1: Table S2). This list was refined by deleting 29 target genes, which were already validated by others using miRTarBase [24] and 4 target genes, which were previously verified by us [19, 20] (Additional file 1: Table S3). Out of the remaining 160 predicted target genes we selected 22 miR-34a-target genes for experimental analysis based on their predicted biological function according to the Gene Ontology (GO) knowledgebase. Figure 1a depicts the detailed affiliation of the target genes in the specialized subcategories of the immune system process category as indicated in the Gene Ontology database.

### Analysis of the predicted miR-34a target genes by dual luciferase assay

**Fig. 2:**
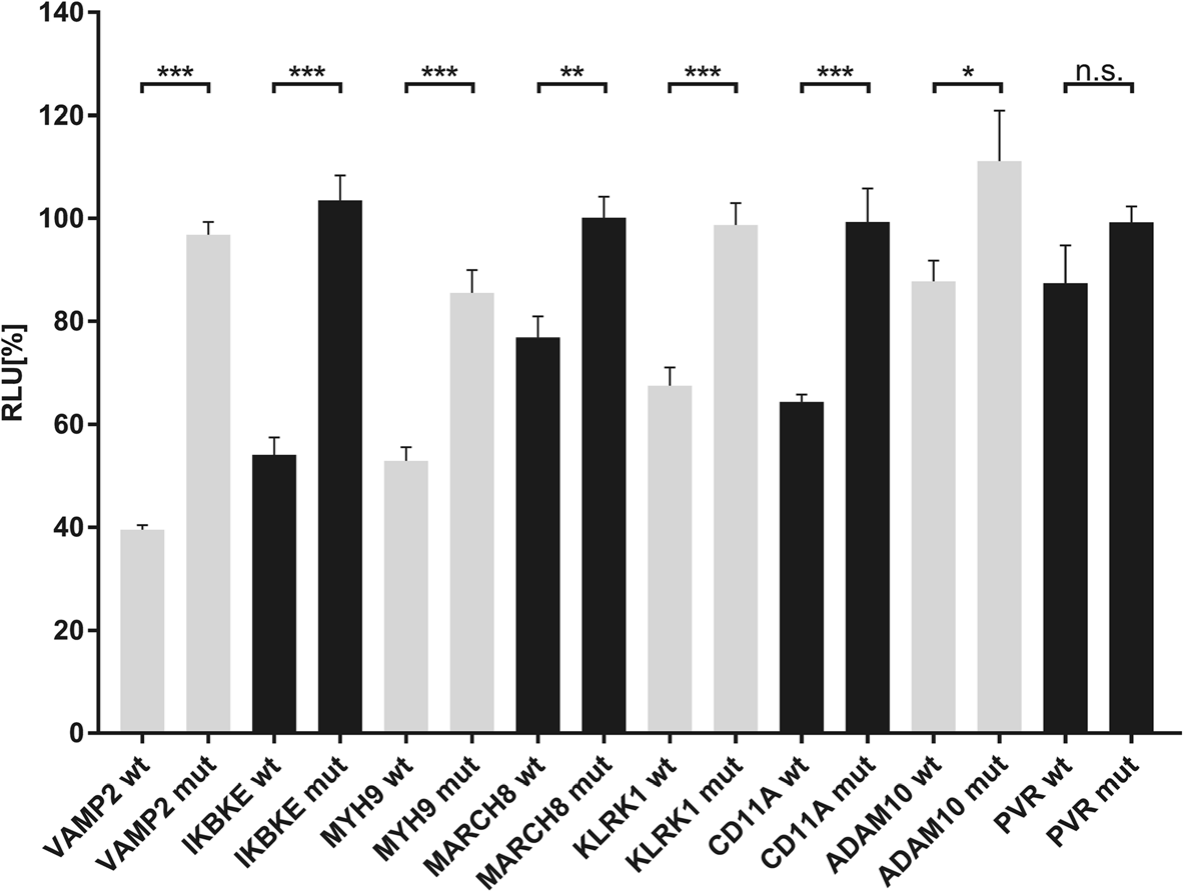
Dual luciferase reporter gene assay of the mutated 3’UTRs of *VAMP2, IKBKE, MYH9, MARCH8, KLRK1, CD11A, ADAM10, and PVR*. HEK 293 T cells were co-transfected with the miR-34a and the wild type reporter plasmids (wt) of the respective target genes or mutated reporter plasmids (mut) of the respective target genes as indicated in the diagram. The luciferase activities were normalized with respect to the luciferase activity of the co-transfection with empty reporter construct. The results represent the mean of four independent experiments carried out in duplicates. Three asterisks correspond to a *p*-value ≤0.001, two asterisks to *p*-value ≤0.01and ≥ 0.001 and one asterisk to a *p*-value ≤0.05 and ≥ 0.01. A *p*-value ≥0.05 is indicated as n.s. (non-significant). Data are represented as mean ± SEM

By our in silico target prediction we identified miR-34a binding sites in the 3′ UTRs of *VAMP2*, *IKBKE*, *MYH9*, *MARCH8*, *KLRK1*, *CD11A*, *TRAFD1*, *CCR1*, *PYDC1*, *PRF1*, *PIK3R2*, *PIK3CD*, *AP1B1*, *ADAM10*, *PVR*, *AP2S1*, *BAD*, *ICOS*, *CD247*, *ZFP36*, *STX8* and *SPN*. The sequences, the positions within the 3’UTRs as well as the types of the miR-34a binding sites are shown in Table 1. We amplified the nucleotides of the miR-34a binding sites by PCR and cloned this PCR product into the pMIR-RNL-TK reporter vector. The cloned reporter constructs were used in a dual luciferase reporter assays. To this end, the reporter plasmids or the empty reporter vector were co-transfected with an empty pSG5 plasmid or a miR-34a expression vector in HEK 293 T cells. The luciferase activities of the co-transfections with reporter constructs harboring the predicted 3’UTRs and miR-34a expression plasmid were normalized with regards to the luciferase activities of the co-transfections with empty reporter vector and miR-34a expression plasmid. We found the strongest reduction of the luciferase activity for the *VAMP2* reporter plasmid that showed an activity of only 49% (*p* value≤0.001) when co-transfected with miR-34a. Likewise, the luciferase activities of the reporter construct for *IKBKE*, *MYH9*, *MARCH8*, *KLRK1*, *CD11A*, *TRAFD1*, *CCR1*, *PYDC1*, *PRF1*, *PIK3R2*, *PIK3CD*, *AP1B1*, *ADAM10*, *PVR*, *AP2S1* and *BAD* were each significantly decreased (Fig. 1b). In detail, the luciferase activity of *IKBKE* reporter vector was decreased to 53%, of *MYH9-* to 54%, of *MARCH8-* to 62%, of *KLRK1-* to 67%, of *CD11A-* to 68%, of *TRAFD1-* to 70%, of *CCR1-* to 71%, of *PYDC1-* to 74%, of *PRF1-* to 76%, of *PIK3R2-* to 78%, of *AP1B1-* to 81%, of *ADAM10-* to 81%, of *PVR-* to 82%, of *AP2S1-* to 90%, and the activity of *BAD-*reporter vector to 91%. The reporter constructs of *ICOS*, *CD247*, *ZFP36*, *STX8* and *SPN* showed no significant reduction of the luciferase activity. To verify the binding of miR-34a to its target sites we mutated all binding sites in the 3’UTRs of *VAMP2*, *IKBKE*, *MYH9*, *MARCH8*, *KLRK1*, *CD11A,* which displayed a distinct decrease of the luciferase activity as well as all binding sites in the 3’UTRs of *ADAM10*, and *PVR,* which showed only a slight reduction. We could validate the direct binding of miR-34a to its binding sites in the 3’UTRs of *VAMP2*, *IKBKE*, *MYH9*, *MARCH8*, *KLRK1*, *CD11A* and *ADAM10* showing a significant increase of the luciferase activity of the mutated reporter constructs in comparison to their wild type 3’UTRs (Fig. 2). For PVR we failed to provide evidence that miR-34a directly binds to its predicted binding site. The dual luciferase assays were done in duplicates and have been repeated 4 times.

**Table 1:**
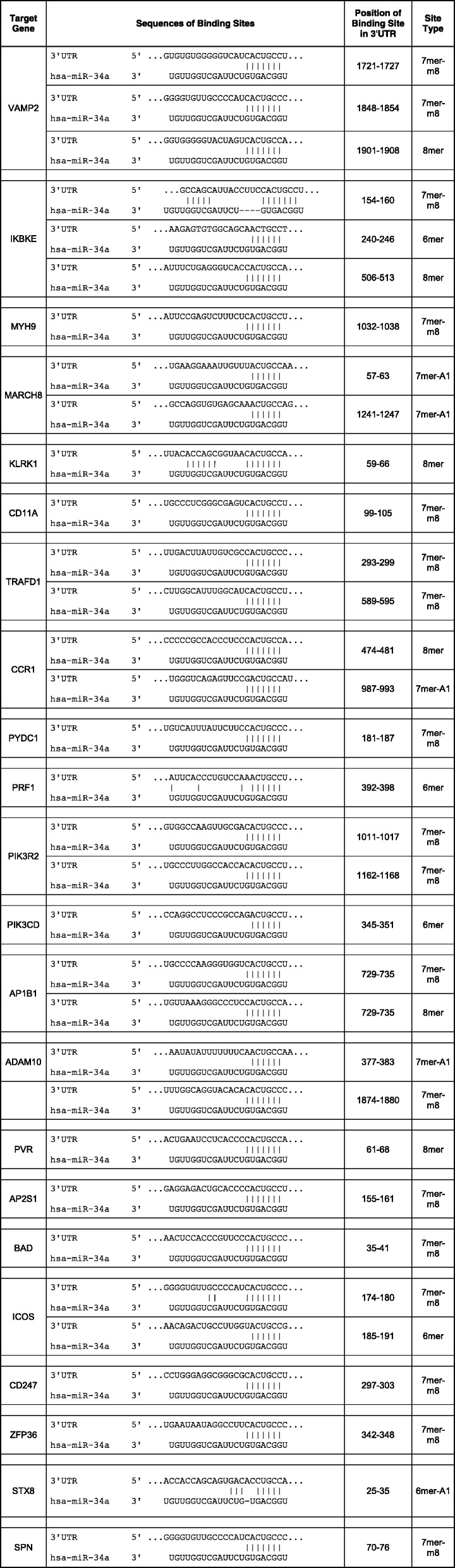
Schematic representation of the reporter gene plasmids

The location of the predicted binding site of miR-34a-5p in respective 3’UTR and additionally the sequences of the binding sites of miR-34a-5p are shown

### Effect of miR-34a over-expression on endogenous protein levels of VAMP2 and CD11A in Jurkat, CD4^+^ T cells and CD8^+^ T cells

**Fig. 3:**
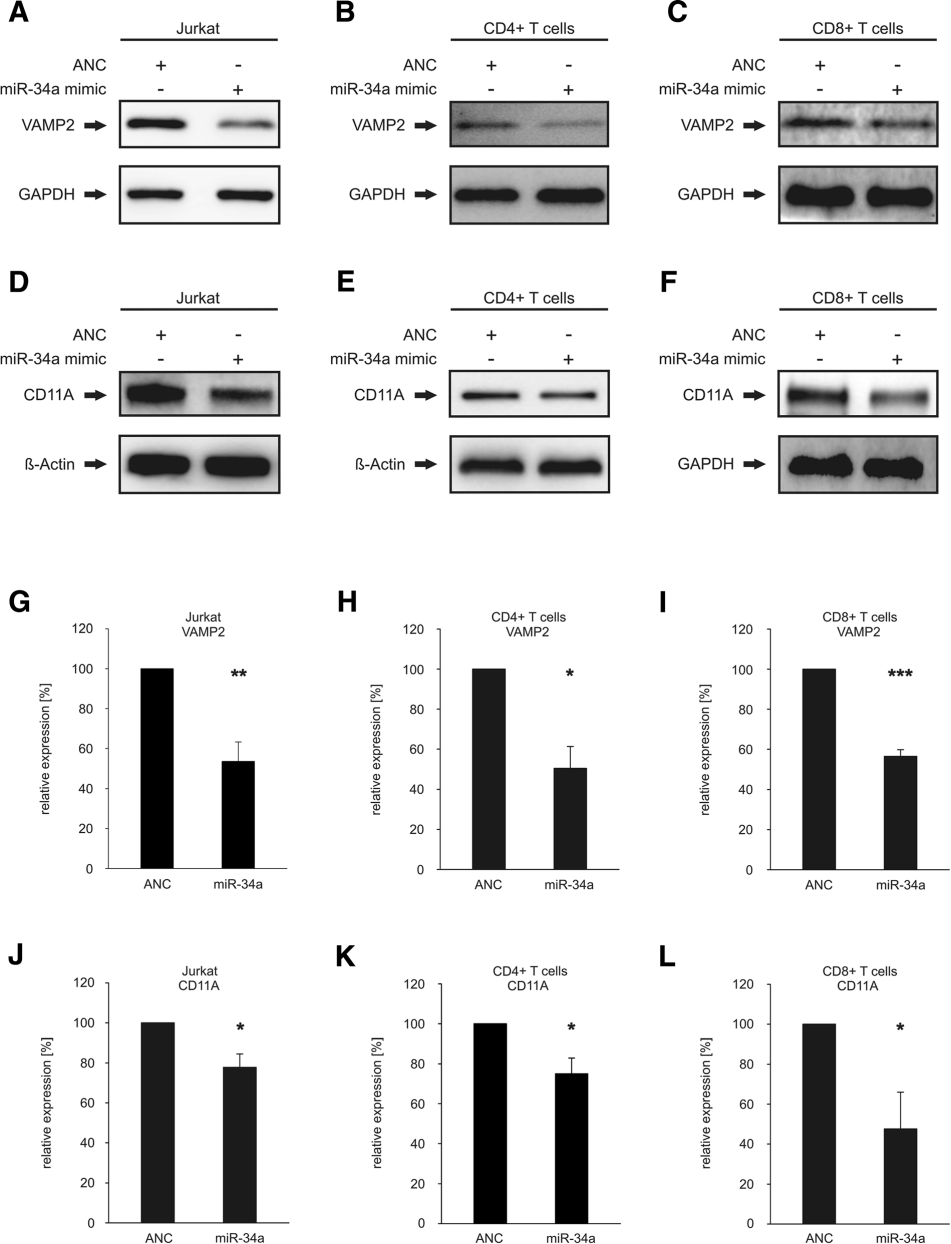
Western blot analysis of VAMP2 and CD11A. **a**-**c** Western blot analysis of VAMP2 in miR-34a transfected Jurkat (**a**), CD4^+^ (**b**) and CD8^+^ T cells (**c**). The cells were transfected either with allstars negative control (ANC) or miR-34a-5p mimic. 48 h after transfection the endogenous protein level of VAMP2 was analyzed by Western blotting using specific antibodies against VAMP2. GAPDH served as loading control. **d**-**f**: Western blot analysis of CD11A in miR-34a transfected Jurkat (**d**), CD4^+^ (**e**) and CD8^+^ T cells (**f**). The cells were transfected either with allstars negative control (ANC) or miR-34a-5p mimic. 48 h after transfection the endogenous protein level of CD11A was analyzed by Western blotting using specific antibodies against CD11A. Beta actin served as loading control in Jurkat cells and CD4^+^ T cells. GAPDH served as loading control in CD8^+^ T cells. g-i: Quantification of endogenous VAMP2 protein levels in miR-34a transfected Jurkat (**g**), CD4^+^ (**h**) and CD8^+^ T cells (**i**). Three independent Western Blots were quantified by densitometry using Image Lab Software. The protein expression of VAMP2 was normalized with respect to the corresponding GAPDH signals of the appropriate samples. One asterisk corresponds to a *p*-value ≤0.05 and ≥ 0.01, two asterisks to *p*-value ≤0.01 and ≥ 0.001 and three asterisks to p-value ≤0.001 . **j**-**l**: Quantification of endogenous CD11A protein levels in miR-34a transfected Jurkat (**j**), CD4^+^ (**k**) and CD8^+^ T cells (**l**). Three independent Western Blots were quantified by densitometry using Image Lab Software. The protein expression of CD11A was normalized with respect to the corresponding beta actin or GAPDH signals of the appropriate samples. One asterisk corresponds to a *p*-value ≤0.05 and ≥ 0.01

We investigated the downstream effect of miR-34a binding to the 3’UTRs of VAMP2 and CD11A on their endogenous protein levels in the Jurkat cell line, in primary CD4^+^ and CD8^+^ T cells. VAMP2 was chosen for further analysis as most affected miR-34a target gene in the dual luciferase assay and CD11A due to its pivotal role in the anti-tumor response and T cell activation. Purity of isolated CD4^+^ and and CD8^+^ T cells were analyzed by flow cytometry (CD4^+^ T cells: mean 91.1% ± 2.5% in three independent experiments, CD8^+^ T cells: mean 91.25% ± 0.9% in three independent experiments). We transfected Jurkat, primary CD4^+^ and CD8^+^ T cells either with “allstars negative control” (ANC) as a non-targeting control or with miR-34a-5p mimic. The over-expression of miR-34a in the transfected CD4^+^ T cells was confirmed by qRT-PCR as shown in Additional file 1: Figure S1. Using specific antibodies against VAMP2 or CD11A we analyzed the endogenous protein levels by Western blotting and found reduced levels of both endogenous VAMP2 and CD11A in the miR-34a transfected Jurkat, CD4^+^ T cells and CD8^+^ T cells (Fig. 3a-f). Representative Western blots out of 3 independent experiments are shown in Fig. 3a-f. Figures 3g-l depict the quantifications of the endogenous VAMP2 and CD11A protein levels for all experiments in Jurkat, CD4^+^ and CD8^+^ T cells. The results show that the mean VAMP2 protein level levels were reduced upon transfection of miR-34a in Jurkat cells to 54% (*p* value≤0.01) (Fig. 3g), in CD4^+^ T cells to 51% (*p* value≤0.05) (Fig. 3h) and in CD8^+^ T cells to 56% (*p* value≤0.001) (Fig. 3i). Mean CD11A protein levels were reduced upon transfection of miR-34a in Jurkat cells to 78% (*p* value≤0.05) (Fig. 3j) and in CD4^+^ T cells to 75% (*p* value≤0.05) (Fig. 3 k) and in CD8^+^ T cells to 48% (*p* value≤0.05) (Fig. 3 l).

**Fig. 4:**
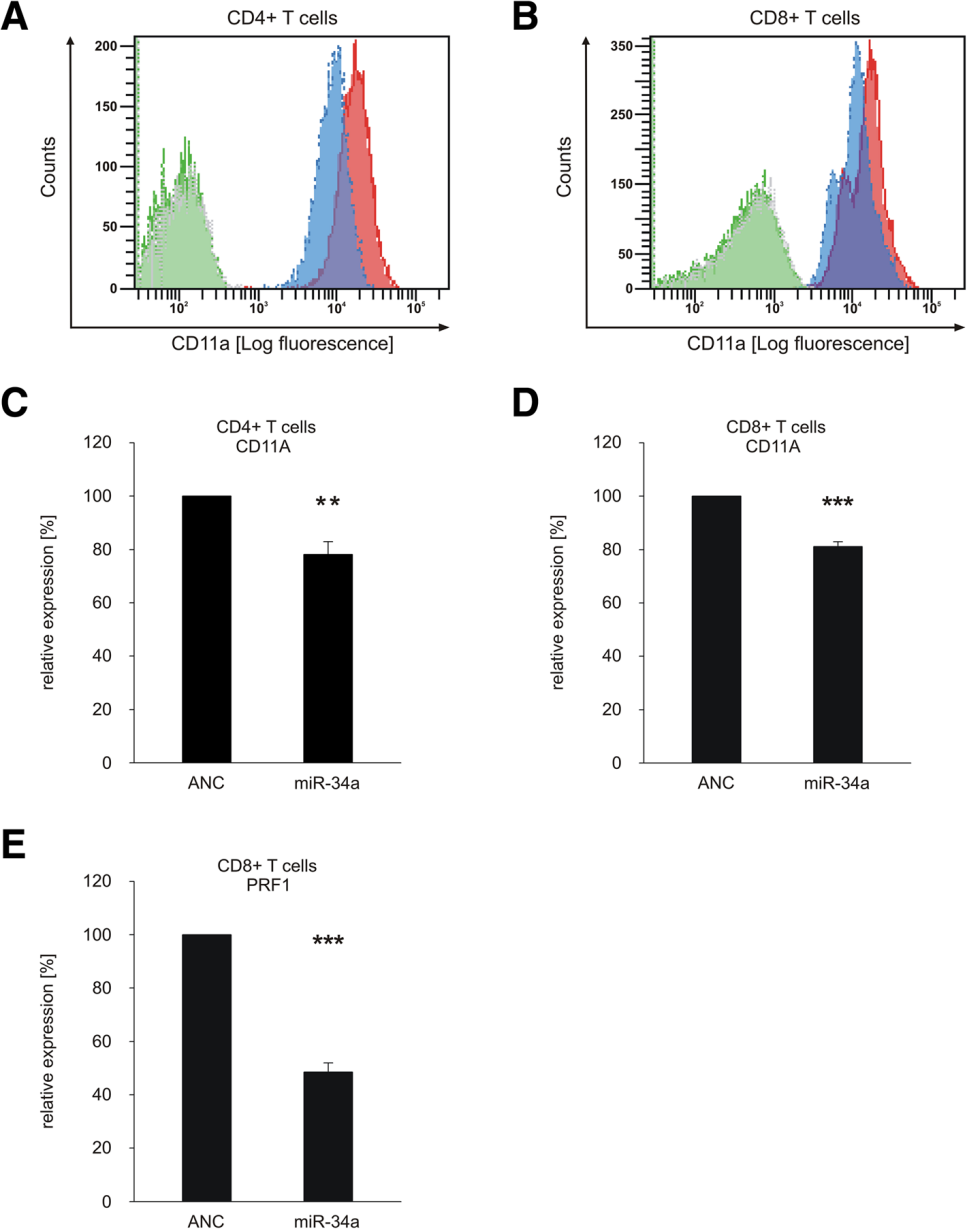
Overexpression of miR-34a reduced cell surface expression of CD11A on primary CD4^+^ or CD8^+^T cells and secretion of PRF1 from CD8^+^ T cells. CD4^+^ or CD8^+^ T cells were transfected either with allstars negative control (ANC) or miR-34a-5p mimic. **a** Mean fluorescence intensities of CD11A expression from ANC-transfected (red) or miR-34a-5p mimic-transfected (blue) CD4^+^ T cells or respective isotype controls (green and grey) was analyzed. **b** Mean fluorescence intensities of CD11A expression from ANC-transfected (red) or miR-34a-5p mimic-transfected (blue) CD8^+^ T cells or respective isotype controls (green and grey) was analyzed. **c** FACS data were summarized from three independent experiments performed in duplicates. Two asterisks correspond to *p*-value ≤0.01 and ≥ 0.001. Data are represented as mean ± SEM. **d** FACS data were summarized from three independent experiments performed in duplicates. Three asterisks correspond to *p*-value ≤0.001. Data are represented as mean ± SEM. **e** ELISA data were quantified from three independent experiments from two different donors. Three asterisks correspond to *p*-value ≤0.001. Data are represented as mean ± SEM

To study the impact of miR-34a overexpression on CD11A cell surface expression in primary CD4^+^ and CD8^+^ T cells we transfected these cells with “allstars negative control” (ANC) or with a miR-34a-5p mimic and analyzed the CD11A expression using flow cytometry (gating strategy is shown in Additional file 1: Figure S2). The analysis of the mean fluorescence intensities of CD11A in CD4^+^ and CD8^+^ T cells showed significantly reduced cell surface levels of CD11A (blue) in comparison to ANC-transfected cells (red) (Fig. 4a +B). Quantification of three independent experiments revealed a decrease of CD11A cell surface expression to 78% (*p* value≤0.01) for CD4^+^ T cells and to 81% (*p* value≤0.001) for CD8^+^ T cells upon transfection with the miR-34a-5p mimic (Fig. 4c +D).

### MiR-34a-5p over-expression in CD8^+^ T cells reduces PRF1 secretion

For functional downstream analysis of miR-34a over-expression in CD8^+^ T cells, we analyzed the PRF1 (Perforin 1) secretion of activated CD8^+^ T cells transfected either with “allstars negative control” (ANC) as a non-targeting control or with miR-34a-5p mimic. Four hours post activation, the secretion of PRF1 of these cells was determined by a PRF1 ELISA. Figure 4 E depicts the quantification of PRF1 in supernatants of control or miR-34a-5p transfected CD8^+^ T cells in three independent experiments from 2 different donors. Mean PRF1 levels decreased upon transfection of miR-34a in CD8^+^ T cells to 49% (*p* value≤0.001) (Fig. 4e).

## Discussion

Recently, we investigated the function of miR-34a in the regulation of signaling pathways in primary T cells and demonstrated the impact of miR-34a over-expression on the modulation of intracellular calcium and NF-κB signaling [19, 20]. Here, we show that miR-34a acts as a major hub in T cell function. Our in silico target prediction combined with a downstream pathway analysis of the predicted miR-34a target genes identified 160 target genes with functions in immune system processes. We selected 22 target genes for further analysis and experimentally verified binding of miR-34a on its predicted target sides in the 3’UTRs of 14 target genes including *VAMP2*, *IKBKE*, *MYH9*, *MARCH8*, *KLRK1*, *CD11A*, *TRAFD1*, *CCR1*, *PYDC1*, *PRF1*, *PIK3R2*, *PIK3CD*, *AP1B1* and *ADAM10*.

IKBKE (inhibitor of nuclear factor kappa B kinase subunit epsilon) represses the activation of NFAT through phosphorylation of NFATc1 during T cell activation. Loss of IKBKE elevates the antiviral and antitumor immunity in mice [25]. This is in contrast to our findings showing that miR-34a over-expression leads to a repression of store-operated Ca^2+^ signaling and impacts downstream calcineurin/NFAT signaling by targeting RCAN1, PPP3R1(Calcineurin), and NFATC4 resulting in a reduction of SOCE and IL-2 secretion [19]. MYH9 (myosin heavy chain 9) is essential for proper formation of the immunological synapse and influences T cell activation [26]. MYH9 is also a central link between cytoskeleton and LFA-1 during T cell migration [27]. One of the main components of LFA-1 is CD11A, which we identified as direct target of miR-34a. Inhibition of these two key components of T cell migration by miR-34a over-expression, which we found in CD3^+^ T cells of lung cancer patients [10], may impact the anti-tumor response by reducing T cell motility. MARCH8 (membrane associated ring-CH-type finger 8) mediates the ubiquitination of mature MHC class II molecules in dentritic cells and B cells while limiting the amount of antigens presented at cell surface to enhance the activation of CD4^+^ thymocytes [28]. Down-regulation of MARCH8 by over-expression of miR-34a could lead to increased amounts of antigens at surface of antigen-presenting cells and attenuate the activation of naïve CD4^+^ T cells. KLRK1 (killer cell lectin like receptor K1 also known as NKG2D) can function as co-stimulatory receptor for the T cell receptor to activate CD8^+^ T cells [29]. A blockade of KLRK1 on CD8^+^ T cells represses the migration over ligand-expressing endothelial cells [30]. Over-expression of miR-34a could impact the activation and motility of CD8^+^ T cells via down-regulation of KLRK1. CD11A (also named ITGAL, integrin subunit alpha L) forms in combination with the common β-chain CD18 the β_2_ integrin LFA-1 heterodimer, which is expressed exclusively on all leukocytes [31]. LFA-1 plays a key role in leukocyte intercellular adhesion through interactions with ICAMs 1–3 [32] and co-stimulation of T cells [33]. The blockade of CD11A by the anti-CD11A antibody efalizumab induced a unique type of T-cell hypo-responsiveness. Although T cells remain fully viable, the direct activation of T cells through different activating receptors (CD2, CD3, CD3/28) is reduced [34]. MiR-34a mediated down-regulation of the LFA-1 subunit CD11A in T cells possibly impacts the anti-tumor immune response in a similar way. PRF1 (perforin 1) is released from secretory granules of cytotoxic T cells in combination with various pro-apoptotic serine protease granzymes [35]. Mutations in *PRF1* lead type 2 FHL (Familial hemophagocytic lymphohistiocytosis) which is a rare, rapidly fatal, autosomal recessive immune disorder characterized by uncontrolled activation of T cells and macrophages and overproduction of inflammatory cytokines [36]. Bi-allelic PRF1 mutations were found in four primary lymphoma patients, who developed cancer beyond the age of 7 years [37]. A down-regulation of PRF1 by miR-34a over-expression in CTLs (cytotoxic T cells) may impact the immune response against cancer cells. PIK3R2 (phosphoinositide-3-kinase regulatory subunit 2) recruits AKT1 and PDPK1 to the cellular membrane activating signaling cascades involve in cell growth, survival, proliferation, motility and morphology [38]. A previous study reported that PIK3R2 limits T cell expansion in mice [39]. PIK3R2 also interacts with the catalytic active PIK3CD (phosphatidylinositol-4,5-bisphosphate 3-kinase catalytic subunit delta) [40], which is implicated in the phosphoinositide 3-kinase δ syndrome (APDS) associated with senescent T cells, lymphadenopathy, and immunodeficiency [41]. Knockdown of both PIK3R2 and PIK3CD by over-expression of miR-34a likely affects the anti-tumor response. ADAM10 (ADAM metallopeptidase domain 10) is a sheddase and catalyzes the secretion of growth factors or cytokines by proteolytic processing of these substrates [42]. Over-expression of ADAM10 in Hodgkin lymphoma resulted in an increased release of NKG2D ligands (NKG2D-L) and reduced activation of effector T lymphocytes [42]. MiR-34a over-expression in CD3+ T cells would reduce the level of ADAM10 and NKG2D ligands resulting in increased activation of effector T lymphocytes. With the receptor of NKG2D ligands KLRK1 (NKG2D) being a direct target of miR-34a, an over-expression of miR-34a may prevent an increased activation of T cells via down-regulation of KLRK1 protein levels. CCR1 (C-C motif chemokine receptor 1) is expressed in a variety of immune cell types like monocytes, CD4^+^ T cells, CD8^+^ T cells, basophils, B cells, eosinophils, neutrophils, natural killer cells, mast cells and dendritic cells and associated with a numerous diseases like multiple sclerosis, rheumatoid arthritis, chronic obstructive pulmonary disease, organ transplantation, Alzheimer’s disease, atherosclerosis and cancer [43]. In radiofrequency ablation-treated tumors of CCR1 deficient mice the loss of CCR1 affects the accumulation of CD11C^+^, CD4^+^, and CD8^+^ T cells in the tumor [44]. VAMP2 (vesicle-associated membrane protein-2, also known as synaptobrevin2) forms together with SNAP25 (synaptosome-associated protein of 25 kD) and STX1A (syntaxin 1A) the SNARE complex between two fusing membranes mediating exocytosis [45]. A study of Matti et al. showed that VAMP2 is responsible for the fusion of lytic granules in cytotoxic T cells [46]. The over-expression of miR-34a in cytotoxic T cells (CTLs) may affect the fusion of lytic granules by down-regulation of VAMP2. The combination of PRF1 down-regulation and the decreased fusion of lytic granules by down-regulation of VAMP2 led to a pronounced repression of PRF1 secretion in miR-34a transfected CD8^+^ T cells. These results support our recent finding that over-expression of miR-34a in CD8^+^ T cells decreases the T cell killing capacity [20].

For the remaining miR-34a targets there is less information on their role in T-cell function: TRAFD1 (TRAF-type zinc finger domain containing 1) expression is inducible by interferon and suppresses Toll-like receptor 4-mediated NF-κB activation [47]. PYDC1 (pyrin domain containing 1) suppresses cytokine mediated NF-κB activation and is found in complex with NCOA6 (nuclear receptor coactivator 6) predominantly in macrophages and granulocytes [48].

AP1B1 (adaptor related protein complex 1 subunit beta 1) mediated protein sorting machinery is crucial for a proper localization of a subset of cytokine receptors in polarized epithelial cells. Deficiency of AP1B1 in mice lead to epithelial immune dysfunction, such as reduced expression of antimicrobial proteins and impaired secretion of immunoglobulin A [49].

## Conclusions

CD8^+^and CD4^+^ T cells, including regulatory T (Treg) and T helper 17 (Th17) T cell subsets, and have increasingly been recognized as key players in carcinogenesis particularly for their role in promotion and maintenance of an immunosuppressive and pro-tumor inflammation environment [50]. Previously we found that miR-34a is strongly induced in the CD3^+^ T cell subpopulation of lung cancer patients [10]. Our previous data and the finding of miR-34a as major hub of translation regulation in immune system processes suggests to utilize miR-34a as target of intervention towards a modulation of the immune responsiveness of T-cells specifically in lung cancer but in also in a broader tumor context.

## Additional file

Additional file 1: **Figure S1.** Analysis of miR-34a-5p over-expression by qRT-PCR. **Figure S2.** FACS Controls. **Table S1.** Sequences of cloning primers.**Table S2.** predicted miR-34a target genes. **Table S3.** validated miR-34a target genes. (PDF 535 kb)

## Data Availability

All data generated or analyzed during this study are included in this published article [and its Additional file 1].

## Abbreviations

3’UTR: 3′ untranslated regions
ADAM10: ADAM metallopeptidase domain 10
ANC: Allstars negative control
AP1B1: Adaptor related protein complex 1 subunit beta 1
APDS: phosphoinositide 3-kinase δ syndrome
CCR1: C-C motif chemokine receptor 1
CD11A: also named ITGAL, integrin subunit alpha L, also known as NKG2D
CTLs: Cytotoxic T cells
FHL: Familial hemophagocytic lymphohistiocytosis
GO: Gene Ontology
IKBKE: Inhibitor of nuclear factor kappa B kinase subunit epsilon
KLRK1: Killer cell lectin like receptor K1
MARCH8: Membrane associated ring-CH-type finger 8
MYH9: Myosin heavy chain 9
NCOA6: Nuclear receptor coactivator 6
PIK3CD: Phosphatidylinositol-4,5-bisphosphate 3-kinase catalytic subunit delta
PIK3R2: Phosphoinositide-3-kinase regulatory subunit 2
PPP3R1: Calcineurin
PRF1: Perforin 1
PYDC1: Pyrin domain containing 1
TCR: T-cell receptor
Th17: T helper 17 cell
TRAFD1: TRAF-type zinc finger domain containing 1
Treg: regulatory T cell
VAMP2: Vesicle-associated membrane protein-2, also known as synaptobrevin2
