# Supplementary material for: miR-34a as hub of T cell regulation networks
Source: J Immunother Cancer. 2019 Jul 16;7:187. doi: 10.1186/s40425-019-0670-5 (PMC6636054; doi:10.1186/s40425-019-0670-5)
Supplement: Supplementary file 1 — Figure S1. Analysis of miR-34a-5p over-expression by qRT-PCR. Figure S2. FACS Controls. Table S1. Sequences of cloning primers.Table S2. predicted miR-34a target genes. Table S3. validated miR-34a target genes. (PDF 535 kb) [file 40425_2019_670_MOESM1_ESM.pdf]

**Supplementary material files**

**Supplementary Figure 1: Analysis of miR-34a-5p over-expression by qRT-PCR.**

Primary $CD4^+$ T cells were transfected either with allstars negative control (ANC) or miR-34a-5p mimic. 48 h post transfection the total RNA was isolated and analyzed by qRT-PCR using a specific hsa-miR-34a-5p primer.

**Supplementary Figure 2: FACS Controls**

$CD4^+$ T cells were stained for CD4 and co-stained for CD11A or the respective isotype controls for 30 min at 4°C. Cells were analyzed by flow cytometry. Gated CD4+ T cells (A) were analyzed for CD11A expression (B, dark grey) in comparison to isotype controls (light grey).

$CD8^+$ T cells were stained for CD8 and co-stained for CD11A or the respective isotype controls for 30 min at 4°C. Cells were analyzed by flow cytometry. Gated $CD8^+$ T cells (C) were analyzed for CD11A expression (D, dark grey) in comparison to isotype controls (light grey).

# Figure S1

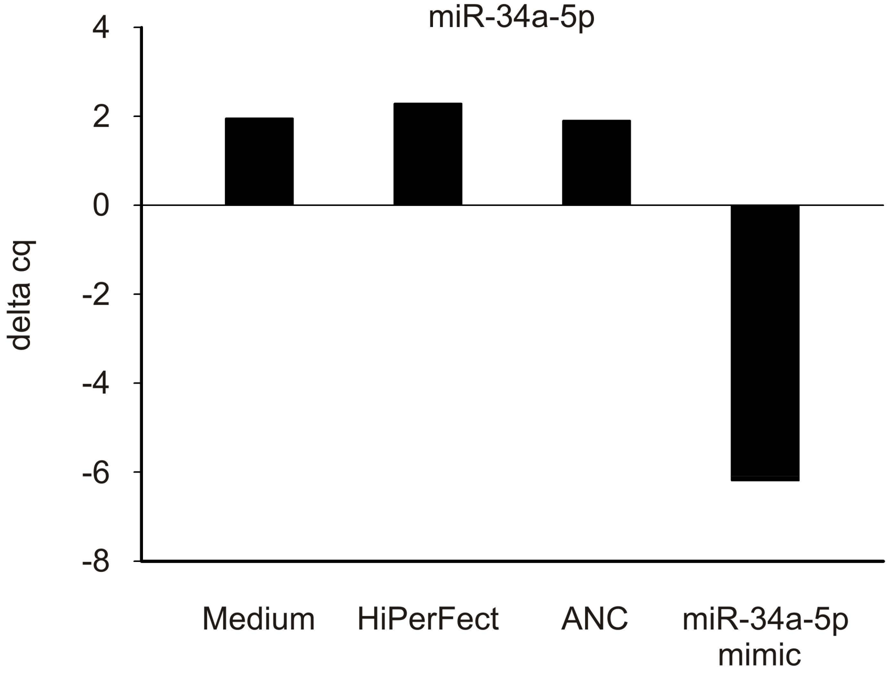
miR-34a-5p
4
2
0
-2
-4
-6
-8
delta cq
Medium
HiPerFect
ANC
miR-34a-5p
mimic

# Figure S2

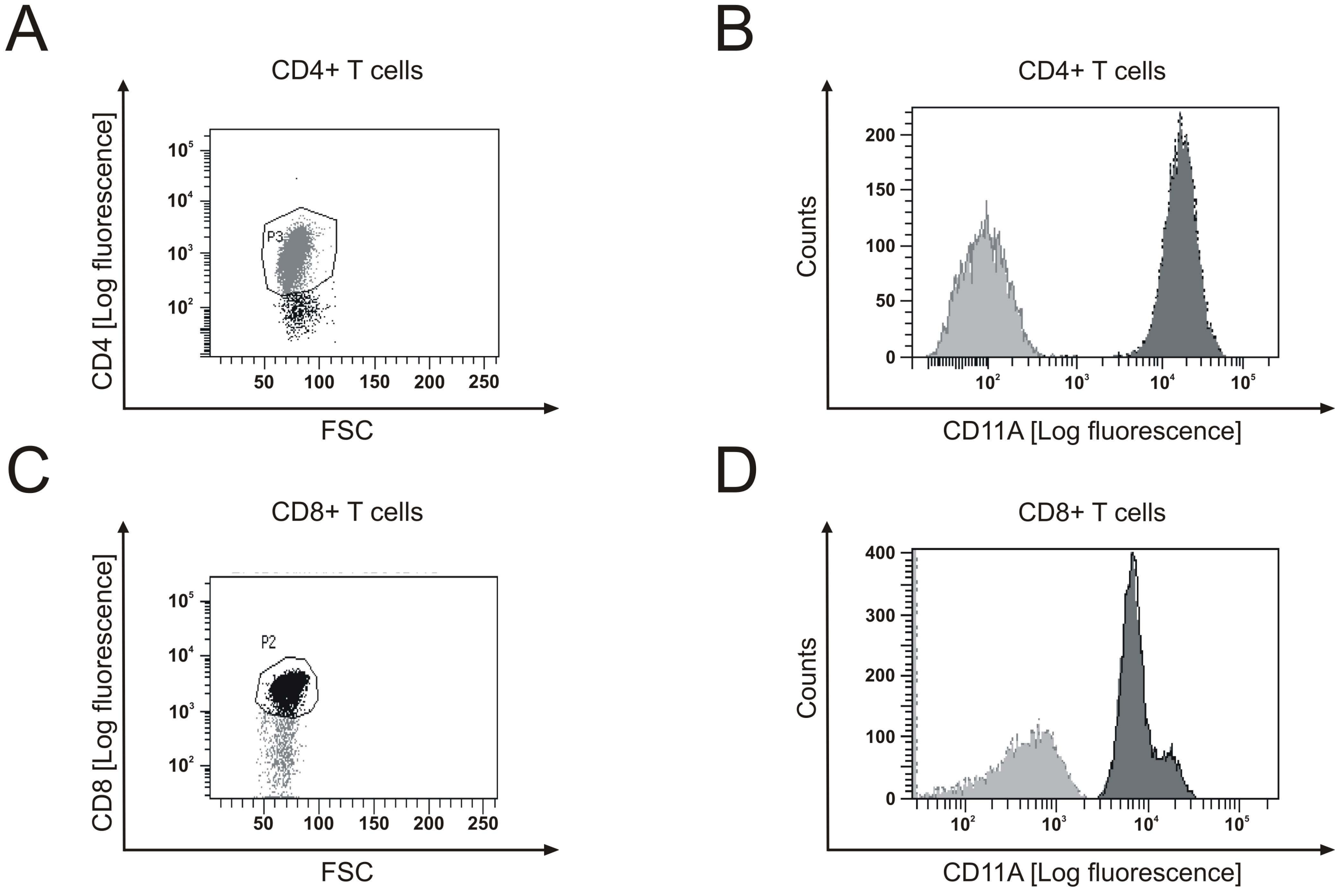

A
CD4+ T cells
CD4 [Log fluorescence]
FSC
P3
B
CD4+ T cells
Counts
CD11A [Log fluorescence]
C
CD8+ T cells
CD8 [Log fluorescence]
FSC
P2
D
CD8+ T cells
Counts
CD11A [Log fluorescence]

# Supplementary Table 1

| target gene | ID | cloning primers | sequence<br>*(restriction sites are underlined)* |
|---|---|---|---|
| VAMP2 | NM_014232.2 | 5'-VAMP2-SpeI<br>3'-VAMP2-NaeI | ggactagtccatcatcctcatcatcatc<br>gccggccctcaatcagttcacccaatgag |
| IKBKE | NM_014002.3 | 5'-IKBKE-SpeI<br>3'-IKBKE-SacI | ggactagtcacatgaggcatcctgaag<br>cgagctcgcatagaaagaacaggaggctc |
| MYH9 | NM_002473.5 | 5'-MYH9-SpeI<br>3'-MYH9-SacI | ggactagtcgaagaggtagatggcaaagc<br>cgagctccctttgtgacagcaactggg |
| MARCH8 | NM_001282866.1 | 5'-MARCH8-SpeI<br>3'-MARCH8-SacI | ggactagtgtgtgcgggttgtcattttc<br>cgagctccagaatgcactgagagtggg |
| KLRK1 | NM_007360.3 | 5'-KLRK1-SpeI<br>3'-KLRK1-SacI | ggactagtgagactgtgcactctatgcctc<br>cgagctcccttcttaactgtgaacctgtg |
| CD11A | NM_002209.2 | 5'-CD11A-SpeI<br>3'-CD11A-SacI | ggactagtgagaaggactctgagagtgg<br>cgagctcctctaactcctgacctcaggtg |
| TRAFD1 | NM_001143906.1 | 5'-TRAFD1-SpeI<br>3'-TRAFD1-SacI | ggactagtcatcggttcctgtcttctgtg<br>cgagctcctgacacttggttttcagagc |
| CCR1 | NM_001295.2 | 5'-CCR1-SpeI<br>3'-CCR1-NaeI | ggactagtctgactcagaccataggagg<br>gccggccctcctacatcaacacagaatc |
| PYDC1 | NM_152901.3 | 5'-PYDC1-SpeI<br>3'-PYDC1-SacI | ggactagtgacatgcgcatgttggag<br>cgagctcccatgaatatttattgggcagtggaagaataaatgacag |
| PRF1 | NM_001083116.1 | 5'-PRF1-SpeI<br>3'-PRF1-SacI | ggactagtgaacagtgagcttggaaaggg<br>cgagctccaaagaagacagagcagctg |
| PIK3R2 | NM_005027.3 | 5'-PIK3R2-SpeI<br>3'-PIK3R2-SacI | ggactagtctgtctctctccatgttggg<br>cgagctccaacgtaccgtacaaaacctg |
| PIK3CD | NM_001350234.1 | 5'-PIK3CD-SpeI<br>3'-PIK3CD-SacI | ggactagtgaccaagcacattggtcc<br>cgagctcggtttcacagagtgctggtc |
| AP1B1 | NM_001127.3 | 5'-AP1B1-SpeI<br>3'-AP1B1-SacI | ggactagtcatcctcaagaactgagacc<br>cgagctcgatctagttaccgtagggtc |
| ADAM10 | NM_001110.3 | 5'-ADAM10-SpeI<br>3'-ADAM10-SacI | ggactagtgtgttccctgttcttcgttg<br>cgagctcccacatcttgattcgcagtgg |
| PVR | NM_006505.4 | 5'-PVR-SpeI<br>3'-PVR-SacI | ggactagtgaccccaatggatgaagac<br>cgagctcgtgggttatttcctgccagtc |
| AP2S1 | NM_001301076.1 | 5'-AP1B1-SpeI<br>3'-AP1B1-SacI | ggactagtgaaacagctgctgatgctac<br>cgagctcgacggcaaggccaggcaggaccacaggtttattgg |
| BAD | NM_004322.3 | 5'-BAD-SpeI<br>3'-BAD-SacI | gactagtcagctggacgcgagtcttcc<br>cgagctccatttggtagtgagcacg |
| ICOS | NM_012092.3 | 5'-ICOS-SpeI<br>3'-ICOS-SacI | ggactagtcatgagagcagtgaacacag<br>cgagctccggtatagtctgacagcaacc |
| CD247 | NM_198053.2 | 5'-CD247-SpeI<br>3'-CD247-NaeI | ggactagtctacctgctggatggaatcc<br>gccggcggtcagtctgttcatcttctgg |
| ZPF36 | NM_003407.3 | 5'-ZPF36-SpeI<br>3'-ZPF36-SacI | ggactagtcgcatctctgtttctgagtg<br>cgagctcccagcttacactcagattg |
| STX8 | NM_004853.2 | 5'-STX8-SpeI<br>3'-STX8-SacI | ggactagtcagaaagtcagcctcttgtg<br>cgagctccagagctttgggggaatttattgagag |
| SPN | NM_001030288.2 | 5'-SPN-SpeI<br>3'-SPN-SacI | ggactagtgtgtcggtgaatagtgaggc<br>cgagctccttcccagactctggtcaac |

**Supplementary Table 2: predicted miR-34a target genes**

| predicted targets in immune system process |
|---|
| DLL1 |
| PRKCQ |
| CACNB3 |
| ARIH2 |
| EFNB1 |
| ESRRA |
| KLRK1 |
| HOXB4 |
| MYH9 |
| SERPINE1 |
| CALCR |
| ADAM10 |
| CDK6 |
| CALCOCO2 |
| NDRG1 |
| LYST |
| CRHR1 |
| PVR |
| DPP4 |
| SWAP70 |
| CXCL10 |
| KIT |
| KITLG |
| NFKBIA |
| NUB1 |
| PKNOX1 |
| PAG1 |
| PURB |
| PVRL1 |
| CCL22 |
| SNCA |
| VCAM1 |
| ZFP36 |
| CAV1 |
| TOB2 |
| FSTL3 |
| CEACAM8 |
| TRAFD1 |
| GPR44 |
| CHRNB2 |
| RASGRP4 |
| CCR1 |
| ANKRD54 |
| CRP |
| CX3CR1 |
| VAMP2 |
| CYP11B1 |

| predicted targets in immune system process |
|---|
| CFD |
| EDN2 |
| RTKN2 |
| ICOSLG |
| DDX58 |
| PYDC1 |
| GBP2 |
| GCH1 |
| B4GALT1 |
| POLL |
| HLA-DQA1 |
| HLX |
| HMGB1 |
| TLX1 |
| TMEM173 |
| AP1B1 |
| IFI35 |
| IKBKB |
| IL1R1 |
| AQP3 |
| IRAK2 |
| ITPKB |
| KCNJ8 |
| ARHGDIB |
| MARCH8 |
| LTF |
| MITF |
| ATM |
| OAS3 |
| GLRX5 |
| PDGFB |
| PML |
| STX8 |
| TOLLIP |
| PRKCA |
| CXCL16 |
| RFX1 |
| BCL6 |
| SMAP1 |
| ROCK1 |
| CXCL11 |
| CXCL5 |
| PRDM16 |
| SELPLG |
| SPN |
| TAL1 |
| TGFBR2 |
| TGFBR3 |
| TLR4 |

| predicted targets in immune system process |
|---|
| WNT2B |
| TFEB |
| ROGDI |
| ICOS |
| NLRC5 |
| TN- |
| FRSF14 |
| BCL10 |
| CCND3 |
| MAP3K14 |
| RSAD2 |
| IL1RL1 |
| CD247 |
| GPR55 |
| CD28 |
| CXCL14 |
| GTPBP1 |
| CD47 |
| CD97 |
| P2RY14 |
| RC3H1 |
| PIK3CD |
| SARM1 |
| IRF1 |
| JARID2 |
| SP2 |
| TACR1 |
| WNT1 |
| BCL2 |
| TRIM10 |
| CPLX2 |
| AP2S1 |
| ADD2 |
| ADK |
| CR2 |
| NFAM1 |
| IL28RA |
| POLR3H |
| FKBP1B |
| JMJD6 |
| IL1RN |
| PIK3R2 |
| IL6R |
| INPP5D |
| KIR2DL1 |
| KIR3DL1 |
| CD46 |
| NOTCH4 |
| FOXP3 |

| predicted targets in immune system process |
| --- |
| ENPP1 |
| PLCG1 |
| SOX6 |
| CTNNBIP1 |
| BAD |
| SATB1 |
| TCF7 |
| THRA |
| C1QC |
| UNG |
| VEGFA |
| XBP1 |
| PRF1 |
| CBFA2T3 |
| ACVR1B |
| CD3E |
| CD86 |
| CD44 |
| ABCC9 |
| SEMA4D |
| MS4A2 |
| SIT1 |
| IRF4 |
| ITGAL |
| JAK2 |
| LY9 |
| LY75 |
| SMAD3 |
| MLL |
| OSM |
| MASP1 |
| ST6GAL1 |
| BMP6 |
| TCF12 |
| TPD52 |
| CALCA |
| ULBP2 |
| IKBKE |
| SH2B3 |
| PATZ1 |
| MAPK1 |
| NOTCH2 |
| PODXL |
| ITCH |
| JAG1 |
| NOTCH1 |
| BCL11B |
| SOX4 |

**Supplementary Table 3: validated miR-34a target genes**

| miRTarBase ID | miRNA | validated Target Gene | Experiments | Support Type | References (PMID) |
|---|---|---|---|---|---|
| MIRT000074 | hsa-miR-34a-5p | JAG1 | Luciferase reporter assay//qRT-PCR//Western blot | Functional MTI | 20351093 |
| MIRT000074 | hsa-miR-34a-5p | JAG1 | Flow//Immunoblot//Luciferase reporter assay//Microarray//qRT-PCR | Functional MTI | 19398721 |
| MIRT000074 | hsa-miR-34a-5p | JAG1 | Flow//In situ hybridization//Luciferase reporter assay//Microarray//qRT-PCR//Western blot | Functional MTI | 24307639 |
| MIRT001010 | hsa-miR-34a-5p | CCND3 | Western blot | Non-Functional MTI | 18406353 |
| MIRT001012 | hsa-miR-34a-5p | CDK6 | qRT-PCR//Luciferase reporter assay//Western blot//Reporter assay | Functional MTI | 18406353 |
| MIRT001012 | hsa-miR-34a-5p | CDK6 | Luciferase reporter assay | Functional MTI | 19461653 |
| MIRT001012 | hsa-miR-34a-5p | CDK6 | qRT-PCR//Western blot//Luciferase reporter assay | Functional MTI | 19584398 |

| miRTarBase ID | miRNA | validated Target Gene | Experiments | Support Type | References (PMID) |
|---|---|---|---|---|---|
| MIRT001012 | hsa-miR-34a-5p | CDK6 | Luciferase reporter assay//Western blot//Microarray | Functional MTI | 17914404 |
| MIRT001012 | hsa-miR-34a-5p | CDK6 | Luciferase reporter assay//qRT-PCR//Western blot | Functional MTI | 19773441 |
| MIRT001012 | hsa-miR-34a-5p | CDK6 | Western blot;Other | Functional MTI | 21240262 |
| MIRT001012 | hsa-miR-34a-5p | CDK6 | qRT-PCR//Western blot | Functional MTI | 23035210 |
| MIRT001012 | hsa-miR-34a-5p | CDK6 | PAR-CLIP | Functional MTI (Weak) | 23592263 |
| MIRT001231 | hsa-miR-34a-5p | WNT1 | Luciferase reporter assay | Functional MTI | 19336450 |
| MIRT001231 | hsa-miR-34a-5p | WNT1 | Flow//Immunoblot//Luciferase reporter assay//Microarray//qRT-PCR | Functional MTI | 19398721 |
| MIRT001231 | hsa-miR-34a-5p | WNT1 | Luciferase reporter assay//qRT-PCR//Western blot | Functional MTI | 28199987 |
| MIRT001757 | hsa-miR-34a-5p | DLL1 | Luciferase reporter assay | Functional MTI | 14697198 |
| MIRT001757 | hsa-miR-34a-5p | DLL1 | Luciferase reporter assay | Functional MTI | 19461653 |
| MIRT001757 | hsa-miR-34a-5p | DLL1 | Luciferase reporter assay | Functional MTI | 20144220 |
| MIRT001757 | hsa-miR-34a-5p | DLL1 | Luciferase reporter assay//qRT-PCR//Western blot | Functional MTI | 23327670 |

| miRTarBase ID | miRNA | validated Target Gene | Experiments | Support Type | References (PMID) |
|---|---|---|---|---|---|
| MIRT002298 | hsa-miR-34a-5p | BCL2 | Luciferase reporter assay//qRT-PCR//Western blot | Functional MTI | 18803879 |
| MIRT002298 | hsa-miR-34a-5p | BCL2 | Luciferase reporter assay//qRT-PCR//Western blot//Reporter assay;Western blot;qRT-PCR;Other | Functional MTI | 19683563 |
| MIRT002298 | hsa-miR-34a-5p | BCL2 | qRT-PCR//Western blot//Luciferase reporter assay | Functional MTI | 18505919 |
| MIRT002298 | hsa-miR-34a-5p | BCL2 | Luciferase reporter assay | Functional MTI | 19461653 |
| MIRT002298 | hsa-miR-34a-5p | BCL2 | Luciferase reporter assay//Western blot//Microarray | Functional MTI | 17914404 |
| MIRT002298 | hsa-miR-34a-5p | BCL2 | Luciferase reporter assay//Microarray//Western blot | Functional MTI | 17656095 |
| MIRT002298 | hsa-miR-34a-5p | BCL2 | Flow//Immunoblot//Luciferase reporter assay//Reporter assay | Functional MTI | 20598588 |
| MIRT002298 | hsa-miR-34a-5p | BCL2 | Reporter assay;Other | Functional MTI | 21399894 |
| MIRT002298 | hsa-miR-34a-5p | BCL2 | Proteomics | Functional MTI (Weak) | 21566225 |
| MIRT002298 | hsa-miR-34a-5p | BCL2 | qRT-PCR//Western blot | Functional MTI | 24565525 |
| MIRT002298 | hsa-miR-34a-5p | BCL2 | Luciferase reporter assay | Functional MTI | 23862748 |

| miRTarBase ID | miRNA | validated Target Gene | Experiments | Support Type | References (PMID) |
|---|---|---|---|---|---|
| MIRT002298 | hsa-miR-34a-5p | BCL2 | qRT-PCR | Functional MTI (Weak) | 23292172 |
| MIRT002298 | hsa-miR-34a-5p | BCL2 | qRT-PCR | Functional MTI (Weak) | 26802970 |
| MIRT002298 | hsa-miR-34a-5p | BCL2 | qRT-PCR//Western blot | Functional MTI | 27939626 |
| MIRT002298 | hsa-miR-34a-5p | BCL2 | Flow//QRTPCR//Western blot | Functional MTI | 26406332 |
| MIRT002298 | hsa-miR-34a-5p | BCL2 | Luciferase reporter assay//qRT-PCR//Western blot | Functional MTI | 25910896 |
| MIRT002875 | hsa-miR-34a-5p | NOTCH1 | Luciferase reporter assay | Functional MTI | 14697198 |
| MIRT002875 | hsa-miR-34a-5p | NOTCH1 | Microarray | Functional MTI (Weak) | 19461653 |
| MIRT002875 | hsa-miR-34a-5p | NOTCH1 | Luciferase reporter assay//qRT-PCR//Western blot | Functional MTI | 20351093 |
| MIRT002875 | hsa-miR-34a-5p | NOTCH1 | Luciferase reporter assay//qRT-PCR//Western blot | Functional MTI | 19773441 |
| MIRT002875 | hsa-miR-34a-5p | NOTCH1 | Luciferase reporter assay//Microarray | Functional MTI | 17150773 |
| MIRT002875 | hsa-miR-34a-5p | NOTCH1 | Immunofluorescence//Immunohistochemistry//Luciferase reporter assay//Microarray//qRT-PCR//Western blot | Functional MTI | 22363487 |

| miRTarBase ID | miRNA | validated Target Gene | Experiments | Support Type | References (PMID) |
|---|---|---|---|---|---|
| MIRT002875 | hsa-miR-34a-5p | NOTCH1 | Luciferase reporter assay//qRT-PCR//Western blot | Functional MTI | 22684561 |
| MIRT002875 | hsa-miR-34a-5p | NOTCH1 | Luciferase reporter assay | Functional MTI | 23085450 |
| MIRT002875 | hsa-miR-34a-5p | NOTCH1 | Western blot | Functional MTI | 23226240 |
| MIRT002875 | hsa-miR-34a-5p | NOTCH1 | Microarray//Western blot | Functional MTI | 22438124 |
| MIRT002875 | hsa-miR-34a-5p | NOTCH1 | qRT-PCR//Western blot | Functional MTI | 23035210 |
| MIRT002875 | hsa-miR-34a-5p | NOTCH1 | qRT-PCR//Western blot | Functional MTI | 23430952 |
| MIRT002875 | hsa-miR-34a-5p | NOTCH1 | Luciferase reporter assay//Western blot | Functional MTI | 28145431 |
| MIRT002875 | hsa-miR-34a-5p | NOTCH1 | Luciferase reporter assay//qRT-PCR//Western blot | Functional MTI | 26493107 |
| MIRT002875 | hsa-miR-34a-5p | NOTCH1 | ELISA//Flow//Luciferase reporter assay//qRT-PCR//Western blot | Functional MTI | 28129650 |
| MIRT004513 | hsa-miR-34a-5p | VEGFA | ELISA//Luciferase reporter assay | Non-Functional MTI | 18320040 |
| MIRT005029 | hsa-miR-34a-5p | NOTCH2 | Luciferase reporter assay//qRT-PCR//Western blot | Functional MTI | 19773441 |

| miRTarBase ID | miRNA | validated Target Gene | Experiments | Support Type | References (PMID) |
|---|---|---|---|---|---|
| MIRT005029 | hsa-miR-34a-5p | NOTCH2 | Immunoblot//Immunocytochemistry//Luciferase reporter assay//Microarray//qRT-PCR//Western blot | Functional MTI | 22363487 |
| MIRT005480 | hsa-miR-34a-5p | CD44 | Immunohistochemistry//qRT-PCR//Western blot//Reporter assay;Other | Functional MTI | 21240262 |
| MIRT005480 | hsa-miR-34a-5p | CD44 | Luciferase reporter assay//qRT-PCR//Western blot | Functional MTI | 24423412 |
| MIRT005480 | hsa-miR-34a-5p | CD44 | qRT-PCR//Western blot | Functional MTI | 25572695 |
| MIRT005480 | hsa-miR-34a-5p | CD44 | Flow//qRT-PCR//Western blot | Functional MTI | 27497057 |
| MIRT005480 | hsa-miR-34a-5p | CD44 | qRT-PCR//Western blot | Functional MTI | 26231042 |
| MIRT005480 | hsa-miR-34a-5p | CD44 | Luciferase reporter assay//qRT-PCR//Western blot | Functional MTI | 25551284 |
| MIRT006286 | hsa-miR-34a-5p | ULBP2 | Flow//Luciferase reporter assay//Western blot | Functional MTI | 22102694 |
| MIRT006286 | hsa-miR-34a-5p | ULBP2 | PAR-CLIP | Functional MTI (Weak) | 26701625 |
| MIRT006869 | hsa-miR-34a-5p | CCL22 | Luciferase reporter assay | Functional MTI | 22975373 |
| MIRT025289 | hsa-miR-34a-5p | EFNB1 | Proteomics | Functional MTI (Weak) | 21566225 |

| miRTarBase ID | miRNA | validated Target Gene | Experiments | Support Type | References (PMID) |
|---|---|---|---|---|---|
| MIRT025289 | hsa-miR-34a-5p | EFNB1 | PAR-CLIP | Functional MTI (Weak) | 23592263 |
| MIRT025369 | hsa-miR-34a-5p | PLCG1 | Proteomics | Functional MTI (Weak) | 21566225 |
| MIRT025409 | hsa-miR-34a-5p | NDRG1 | Proteomics | Functional MTI (Weak) | 21566225 |
| MIRT025584 | hsa-miR-34a-5p | TPD52 | Sequencing | Functional MTI (Weak) | 20371350 |
| MIRT025584 | hsa-miR-34a-5p | TPD52 | Proteomics | Functional MTI (Weak) | 21566225 |
| MIRT307050 | hsa-miR-34a-5p | TGFBR2 | PAR-CLIP | Functional MTI (Weak) | 22012620 |
| MIRT437939 | hsa-miR-34a-5p | ARHGDIB | Western blot//Luciferase reporter assay// | Functional MTI | 23349340 |
| MIRT438239 | hsa-miR-34a-5p | KIT | Luciferase reporter assay//Western blot | Functional MTI | 24009080 |
| MIRT438239 | hsa-miR-34a-5p | KIT | Luciferase reporter assay | Functional MTI | 27056900 |
| MIRT438704 | hsa-miR-34a-5p | IL6R | ChIP-seq//Immunofluorescence//qRT-PCR//Western blot | Functional MTI | 24642471 |
| MIRT438704 | hsa-miR-34a-5p | IL6R | Luciferase reporter assay//qRT-PCR//Western blot | Functional MTI | 25973020 |
| MIRT438704 | hsa-miR-34a-5p | IL6R | Luciferase reporter assay | Functional MTI | 26091352 |
| MIRT535098 | hsa-miR-34a-5p | PODXL | PAR-CLIP | Functional MTI (Weak) | 22012620 |

| miRTarBase ID | miRNA | validated Target Gene | Experiments | Support Type | References (PMID) |
|---|---|---|---|---|---|
| MIRT702573 | hsa-miR-34a-5p | JARID2 | HITS-CLIP | Functional MTI (Weak) | 23313552 |
| MIRT731117 | hsa-miR-34a-5p | TCF7 | qRT-PCR//Luciferase reporter assay//Immunohistochemistry//Western blot | Functional MTI | 25436980 |
| MIRT732534 | hsa-miR-34a-5p | CACNB3 | Immunocytochemistry//Luciferase reporter assay//Western blot | Functional MTI | 25623948 |
| MIRT732593 | hsa-miR-34a-5p | MAP3K14 | qRT-PCR | Functional MTI (Weak) | 25864118 |
| MIRT733801 | hsa-miR-34a-5p | NLRC5 | Luciferase reporter assay//qRT-PCR//Western blot | Functional MTI | 27423514 |
| MIRT733971 | hsa-miR-34a-5p | BCL10 | PCR array | Functional MTI (Weak) | 28097098 |
| MIRT734565 | hsa-miR-34a-5p | FKBP1B | Luciferase reporter assay | Functional MTI | 26471303 |
| MIRT741440 | hsa-miR-34a-5p | POLL | PAR-CLIP | Functional MTI (Weak) | 26701625 |
| miR-34a target genes validated by Hart et al. | | | | | |
| n.a. | hsa-miR-34a-5p | PRKCQ | Luciferase reporter assay//Western blot | Functional MTI | 27144431 |
| n.a. | hsa-miR-34a-5p | PRKCA | Luciferase reporter assay//Western blot | Functional MTI | 27144431 |
| n.a. | hsa-miR-34a-5p | NFKBIA | Luciferase reporter assay//Western blot | Functional MTI | 30718475 |
| n.a. | hsa-miR-34a-5p | CD3E | Luciferase reporter assay//FACS | Functional MTI | 30718475 |
